# Characterization of crystalline cellulose of jute reinforced poly (vinyl alcohol) (PVA) biocomposite film for potential biomedical applications

**DOI:** 10.1007/s40204-014-0023-x

**Published:** 2014-04-03

**Authors:** Mohammed Mizanur Rahman, Sanjida Afrin, Papia Haque

**Affiliations:** Department of Applied Chemistry and Chemical Engineering, University of Dhaka, Dhaka, 1000 Bangladesh

**Keywords:** Jute, Crystalline cellulose, Biocomposite, Wound healing, Reinforcing agent, PVA

## Abstract

Cellulose crystals (CC) were chemically derived from jute by alkali treatment, bleaching and subsequent hydrolysis with 40 % sulfuric acid. Infrared spectroscopy (FT-IR) suggested sufficient removal of lignin and hemicellulose from the raw jute and scanning electron microscopy (SEM), and X-ray diffraction (XRD) studies demonstrated the preparation of microcrystalline cellulose. CC reinforced polyvinyl alcohol (PVA) composite was prepared by solution casting method under laminar flow. In order to maintain uniform dispersion of 3–15 % (w/w) of the CC in the composite *N, N* dimethylformamide (DMF) was used as a dispersant. FT-IR, XRD, SEM, thermogravimetric analysis (TG, DTG and DTA) and thermomechanical analyses (TMA) were used to characterize the CC and the composites. The study of tensile properties showed that tensile strength (TS) and modulus (TM) increase with increasing CC content up to 9 % and then decrease with the addition of a high content of CC (above 9 %) because of the aggregation of CCs in the composite. The highest TS (43.9 MPa) and TM (2,190 MPa) have been shown to be the composite prepared with 9 % CC and the lowest to be from pure PVA film 17.1 and 1470 MPa. In addition, the composites have showed no cytotoxicity that can also prohibit microbial growth and; hence, it can be a potential material for biomedical applications such as wound healing accelerators.

**Electronic supplementary material:**

The online version of this article (doi:10.1007/s40204-014-0023-x) contains supplementary material, which is available to authorized users.

## Introduction

The use of natural fibers as reinforcements in polymers and composites has attracted much attention due to the environmental concerns, availability, renewable feed stocks, relatively low cost and biodegradability. Crystalline cellulose (both nano and microcrystalline) has generated a great deal of interest as a source of micrometer and nanometer sized fillers because of their very good mechanical properties (Azizi Samir et al. 2005). This feature, along with the remarkable suitability to reinforce with different matrices, provides superior mechanical performance and makes it possible to use in a wide range of applications like medical, pharmaceutical, filtration, and catalysis fields, etc. (Kotek 2008). Moreover, due to better mechanical properties, polymer composites with cellulose crystals (CC) are able to substitute glass-fiber-containing composites in some important applications such as in the automotive or construction industries and have found potential applications in biomedical and cosmetic industries, the electrical and electronic field, and the paper and packaging industry (Bledzki and Gassan, 1999; Hoenich 2007; Lee et al. 2009; Mathew et al. 2005).

The production of nano-scale cellulose fibers and their application in composite materials has gained increasing attention in recent times. Considerable research has been done regarding the extraction of CC from different sources and on preparing polymer composites with them (Iwatake et al. 2008; Mathew et al. 2005; Nakagaito and Yano 2008a, b; Özgür Seydibeyoğlu and Oksman 2008). The mechanical and chemical treatments have been the most applied methods to obtain nano/micro CC. Nakagaito and Yano (2008b) obtained cellulose nanofibers from kraft pulp after repeated passes (16–30) through a refiner and prepared a composite with improved mechanical properties based on a phenolic resin reinforced with these fibers. Cellulose whiskers with a length between 200 nm and 400 nm were isolated from microcrystalline cellulose (MCC) by acid hydrolysis using sulphuric acid with a concentration of 63.5 % (Cheng et al. 2007). Jute is another important source of cellulose and the percentage of crystallinity of jute fiber (73.4 %) was considerably higher than that of other non-woods (Jahan et al. 2009). The higher crystallinity of cellulose in jute fiber indicates its suitability in the preparation of micro-/nano-cellulose crystal (MCC/CNCs). One of the drawbacks of using CNCs is their high tendency to agglomerate due to the large number of hydroxyl groups on their surface (highly polar and hydrophilic). This makes dispersion of these crystals very difficult in polymer matrices, especially those that are non-polar or hydrophobic. In this case, the properties of the interfacial zone or interphase can play a major role in overall properties of the cellulose nanocrystal composite materials.

Polyvinyl alcohol (PVA) composites prepared with these cellulose crystals showed significantly improved tensile and thermal properties. PVA is a water-soluble and biodegradable polymer with excellent chemical resistance; as such it is an interesting material for high-tech applications (Zhang et al. 2009). PVA hydrogels exhibit biocompatibility as well as a high elastic modulus even at relatively high-water concentrations. PVA hydrogels have been employed in several biomedical applications, including drug delivery, contact lenses, artificial organs, wound healing, cartilage, etc. (Peppas and Mongia, 1997; Tan and Saltzman 2004). PVA has also been proposed as a promising biomaterial to replace diseased or damaged articular cartilage. However, it has limited durability and does not adhere well to tissue. For example, for articular cartilage applications, PVA may require the use of a fixation method for better adhesion (Kobayashi et al. 2003). In the area of the skin scaffold, PVA needs to be compounded with some other filling materials having bioactivity and, in this case, nano filler or nano reinforcing agents will have significant impact on its overall physico-mechanical properties. For many other applications, the mechanical properties of PVA can be substantially improved without damaging its other valuable properties such as transparency and flexibility.

The main goal of this work is to extract CC from jute and process optimization and its characterization and application as a reinforcing agent to prepare biocomposites with PVA. Although there has been some research work done on the preparation of micro crystalline cellulose-PVA composites from cellulose crystals of different sources such as bagasse, wood, cotton, sisal; etc., there has been no such report in the literature which addresses the application of CC of jute for biocomposites with PVA. In addition, the composites were described by different characterization processes such as thermal, morphological, structural, hardness etc. but there were no biochemical studies for the composites. In the present study, micro crystalline cellulose are prepared from jute by sulfuric acid hydrolysis of mercerized and bleached jute fiber and the crystals were used to reinforce biocomposite scaffold/film with PVA by the solution casting method. The research also addresses the optimization of the content of CC in the composites and complete evaluation of chemical, thermal, mechanical and biochemical activities of the composite materials. Different physic-mechanical, structural, thermal, morphological and in vitro biochemical properties were evaluated with respect to CC loading. Furthermore, this work will describe the possibility of the application of the composite in skin tissue engineering.

## Experimental

### Materials

White jute (*Corchorus capsularis*) was purchased from the local market of Tangail, Bangladesh. PVA (C_2_H_4_O)_n_ was obtained from Qualikems fine chem Pvt. Ltd, Delhi, India having the degree of polymerization of 1,700–1,800.

### Methods

#### Extraction of cellulose

Jute fibers were subjected to a washing pre-treatment to remove impurities and waxy substances covering the external surface of fiber cell walls. The fibers were cut into small size (about 2 cm) by using scissors and then milled into fine size by using a mechanical milling machine. These fibers (25 g) were dispersed in distilled water (500 mL) for 10 min at room temperature and stirred for 2 h at 50 °C using a glass rod and filtered in order to remove soluble extractives in water. The dried fiber was mercerized with 2 % NaOH solution at 80 °C for 6 h with mechanical stirring followed by thorough washing until neutralized and drying. The dried fibers were then bleached with 2 wt.% NaClO_2_ at 80 °C for 4 h with mechanical stirring, washed and dried in an oven. The bleached fibers were further treated in a concentrated sulfuric acid solution (40 wt% sulfuric acid in water) at 45 °C for 10 h with mechanical stirring. The ratio of fibers to acid solution was 1:15 g L^−1^. After the treatment, the hydrolyzed cellulose samples are neutralized by 30 wt% NaOH solution in water, and then the crystals were washed for four times. After each washing step, the crystals were separated from the solution by centrifugation at 8,000 rpm for 10 min. Finally, the CC were obtained after a freeze drying for 48 h.

#### Preparation of CC-reinforced PVA film

PVA-CC biocomposites with various filler contents were prepared by mixing the various amounts of CCs in PVA as shown in Table 1 using a magnetic stirrer and ultrasonication. Prior to this, the different amounts of CC according to Table 1 were dispersed well in each of 5.0 mL of *N*,*N*-dimethyl formamide (DMF) solution.DMF worked as a dispersant and helped for homogeneous mixing of CC with PVA for the preparation of final composites. With constant stirring, these suspensions were poured into different PVA solutions for different compositions of the composites. The stirring was performed at 80 °C for 90 min. The mixture was cooled at room temperature, then cast on a silicone rubber sheet and placed under a laminar flow for 2 days until they were completely dried. The dried films were stored in desiccators for further use.

**Table 1:** Composition of the CC/PVA composites

Sample no.	Composite film	Amount of cellulose crystals (g)	Amount of PVA (g)
A	Film with 0 % CC	0.0	5.00
B	Film with 3 % CC	0.15	4.85
C	Film with 6 % CC	0.30	4.70
D	Film with 9 % CC	0.45	4.55
E	Film with 12 % CC	0.60	4.40
F	Film with 15 % CC	0.75	4.25

#### Fourier transform infrared spectroscopy

Fourier transform infrared spectroscopy was used to trace any changes in the chemical structure of the CC and CC reinforced PVA composites. The FTIR spectra were recorded with a ATR-FTIR spectrophotometer (Model-01831, SHIMADZU Corp. Japan). The samples were prepared by mixing the samples with KBr and compressing the mixture into the disk. The spectra were obtained at a resolution of 4 cm^−1^ in the range 4,000–400 cm^−1^.

#### Surface morphology

The morphology of the CCs and the bio-composites were checked using scanning electron microscopy (SEM) using a JEOL JSM 6490A, Japan microscope operated with an accelerating voltage of 5 kV. A small portion of the fibers or films were fixed on conductive carbon tape and mounted on the support and then sputtered with an approximately 5 nm layer of graphite.

#### X-ray diffraction and crystallinity measurement

The crystallinity of the cellulose fibre was examined by using a X-ray diffractometer (Model JDX-8P, JEOL Ltd., Tokyo, Japan) using CuK_α_ radiation of wavelength, *λ* = 1.5418 Å. The diffracted intensity of Cu K_α_ radiation was assessed at a voltage of 40 kV and 30 mA. The samples were dried and measured in a 2*θ* range between 5°–30°. Crystallinity was commonly measured as a ratio between the diffraction portion from the crystalline part of the sample, *A*_c_, and the total diffraction from the same sample, *A*_total_. The values of *A*_c_ could be obtained after an appropriate subtraction of the scattering portion from the background, *A*_b_. The relative crystallinity index was calculated by Eq. (1) (Alemdar and Sain 2008) as follows:

1 $$\text{Crystallanity (}\%)=(A_{\text{c}}\times 100)/\;A_{\text{total}},$$

2 $$A_{\text{total}}=A_{\text{c}}+A_{\text{b}}.$$

#### Thermogravimetric analysis (TG/DTA/DTG)

Thermogravimetry (TG), differential thermal analysis (DTA) and differential thermogravimetry (DTG) of cellulose and the composites were performed by using a TG/DTA EXTAR 6000 STATION, Seiko Instruments Inc. Japan. Samples of about 2.5 mg kept in a aluminum cell were heated in the temperature range of 30–600 °C at a heating rate of 10 °C/min under nitrogen atmosphere.

#### Thermomechanical analysis (TMA)

Thermo-mechanical analysis of films was carried out by using a Shimadzu TMA-50, Japan, analyzer. The samples (size 4 mm × 4 mm) were cut and placed in an aluminium crucible and a lid was placed over the sample under a constant load of 100 mN. The sample was heated from 20 to 140 °C at a heating rate of 5 °C s^−1^ under nitrogen atmosphere.

#### Mechanical properties

Tensile strength (TS) and percent elongation at break (Eb) of the composites were measured by a Universal Testing Machine (Hounsfield, Model H50 Ks0404, UK) following ASTM D3039 having efficiency within ±1 %. The machine speed was 100 mm min^−1^ with gauze length and load of 8 cm and 500 N, respectively. Seven different composites with different concentrations of CC in PVA were analyzed. The composite films were cut into a rectangular size with the dimension of 10 cm × 1 cm, and the cut sample was placed into the machine along the length. Tensile properties were measured at 55–60 % relative humidity and minimum eight samples were tested to take an average of any data.

#### Water uptake and moisture content analysis

A water uptake test was performed to observe the water absorption and sustainability in water. Preweighed composite films with various CC loading were taken and soaked in water for a different time period (5–150 min). It was measured by a computerized moisture content analysis machine (KERN RH 120-3, max. 120 g, Germany). After the tests were over, the CC sample (~0.3 g) and composite samples (~0.3 g) were dried at 105 °C to a constant weight and further analyzed.

#### Microbial sensitivity and in vitro cytotoxicity study

Antimicrobial activity of the composites against *Bacillus subtilis*, and *Escherichia coli* were investigated by the disc diffusion method. This method was performed in a Muller Hinton medium. The media used for antimicrobial activity was poured into a sterile petri dish and allowed to cool. Then the test culture (*Bacillus subtilis* and *Escherichia coli*) was inoculated properly onto the media. The samples (A–H numbered by 1–6) were autoclaved for 2 h and 10 m to remove any bacterial contamination. It is important to mention that PVA composites melted a little bit at autoclave temperature though the zone was still possible to identify. The plates were incubated overnight at 37 °C and the inhibition zone was measured in the evaluation of antimicrobial activity of the biocomposites.

In vitro, a cytotoxicity test was performed using brine shrimp lethality bioassay method 1. Brine shrimp (*Artemia salina*) were hatched using brine shrimp eggs in a conical shaped vessel (1 L), filled with sterile artificial seawater, and the pH was adjusted at 8.5 using 0.1 N NaOH under constant aeration for 48 h. After hatching, active nauplii free from egg shells were collected from the brighter portion of the hatching chamber and used for the assay. The composites were dissolved in artificial seawater at 0.20 mg mL^−1^ concentration and were taken in petri dishes where the active nauplii were inoculated. After overnight incubation, the nauplii were counted. The 0.5 mg mL^−1^ of vincristine sulfate (an anticancer drug) was considered as positive control.

## Results and discussions

### Extraction of micro and nano-crystalline cellulose from jute

**Fig. 1:**
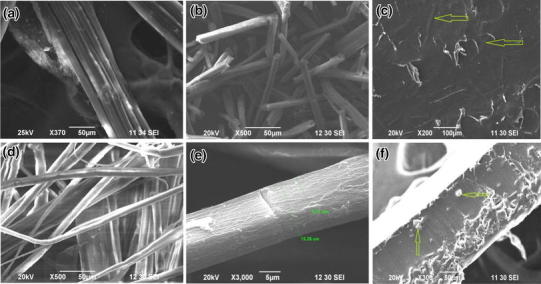
SEM images of cellulose crystals and CC reinforced biocomposites, **a** raw jute, **b** bleached jute fibers, **c**, **d** acid hydrolyzed crystalline jute fibers), **e** 9 % CC reinforced PVA composite and **f** fractured surface of 9 % CC-PVA composite

Micro and nano crystalline cellulose extraction procedure from jute consists of washing, lignin removal, bleaching and acid hydrolysis. When cellulosic fibre is treated with NaOH, it changes the structure of cellulose I to cellulose II by a process known as mercerization (Kotek 2008). Alkali treatment may also remove natural and artificial impurities and produce a rough surface topography as shown in Fig. 1b. Therefore, the mechanical interlocking of the prepared CC with PVA matrix at the interface was expected in the final composites.

The mercerized fiber was washed, dried and then subjected to acid treatment with 40 % sulfuric acid. This happened because acid hydrolysis was effective in dissolving the amorphous cellulose, which ultimately produced micro/nano-crystalline cellulose. It is noteworthy for mention here that several attempts to hydrolyze jute fibers with 64 % acid as mentioned in the literature (Hoenich 2007; Lee et al. 2009) failed and the fibers were obtained as acid burnt due to higher acid concentration.

### Morphological study

The SEM images of raw jute, mercerized jute fibers, CC, and the composites are shown in Fig. 1a–e. It was observed from the figures that the CC appeared in micro rod like structures, and the diameters of micro crystals were in the range of about 5–6 μm, having an aspect ratio (l/*d*) around 6–7 (Fig. 1c, d). Although it was expected there would be some nanocrystals after acid hydrolysis, due to the detection limitation of SEM, they were not visible in any of the images as shown in Fig. 1. We have assumed that the change of structures and size of the fibers have occurred due to the removal of the amorphous regions of the cellulose by acid treatment.

Figure 1e shows the SEM micrograph of the surface of the composite that revealed a smooth and even surface without any porosity and uniform dispersion of CC in the matrix. The fractured surfaces of the composite specimens were studied and shown in Fig. 1f to understand the failure mechanisms and possible interaction between CC and PVA. The fracture surfaces of PVA/CC composites were also observed to be smooth and even, which indicates less amount of bonding between the reinforcement and the matrix.

### FTIR studies

**Fig. 2:**
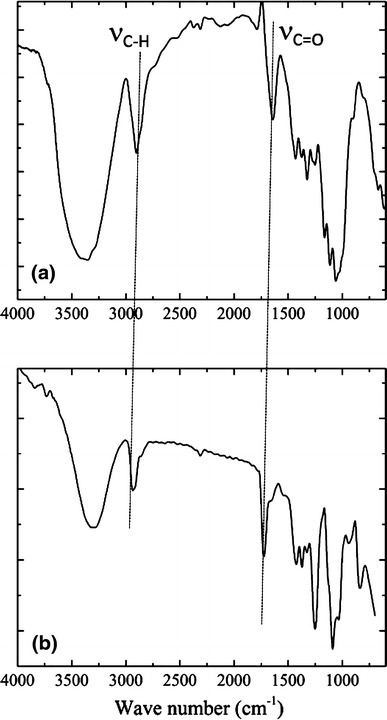
FT-IR spectra of **a** CC and **b** 9 % CC reinforced PVA composites

The FT-IR spectra of CC and its composite sample D are shown in Fig. 2. The band for acetyl and uronic ester groups of the hemicellulose at 1,714 cm^−1^ (Bledzki and Gassan 1999; Mathew et al. 2005) was absent in CC, which indicates that the CC are completely pure. Moreover, there was an absorption at 710 cm^−1^ and a weak shoulder at 750 cm^−1^ due to I_α_ (triclinic) and I_β_ (monoclinic) cellulose structure (Nakagaito and Yano 2008a). The chemical treatment of raw jute for removing the lignin, the absorption bands in 1,730, 1,620, 1,595 and 1,512 cm,^−1^ corresponding to the functional groups of lignin, are not observed on the spectrum of CC (Nakagaito and Yano 2008b). For the composite, the large band observed between 3,600 and 3,200 cm^−1^ is linked to the stretching of O–H from the intramolecular and intermolecular hydrogen bonds, the vibrational band observed between 2,840 and 3,000 cm^−1^ refers to the stretching C–H from alkyl groups and the peaks between 1,750–1,620 cm^−1^ are due to the stretching C=O and C–O due to the formation of ester linkage between PVA and CC. The peaks for C–O–C at 1,150–1,085 cm^−1^ and for C–O at 1,141 cm^−1^, bending vibration related to CH_2_ groups at 1,461–1,417 cm^−1^ (Iwatake et al. 2008), are also observed in the spectrum of the composite. Furthermore, the composite spectra showed that the absorption at 705 cm^−1^ has disappeared from native PVA and the peaks at 512 cm^−1^ and 628–648 cm^−1^ are weakened (as shown in Fig S4 in supporting information). This region was similar to that of the CC spectra. The weakness, disappearance, and shift of the characteristic absorption band might have resulted from the interactions of different OH groups in the PVA and CC molecular chains (Han et al. 2009). This may indicate the development of new inter-molecular and intra-molecular hydrogen bonds and a change in the conformation between PVA and CC (Oh et al. 2005).

### X-RD analysis

**Fig. 3:**
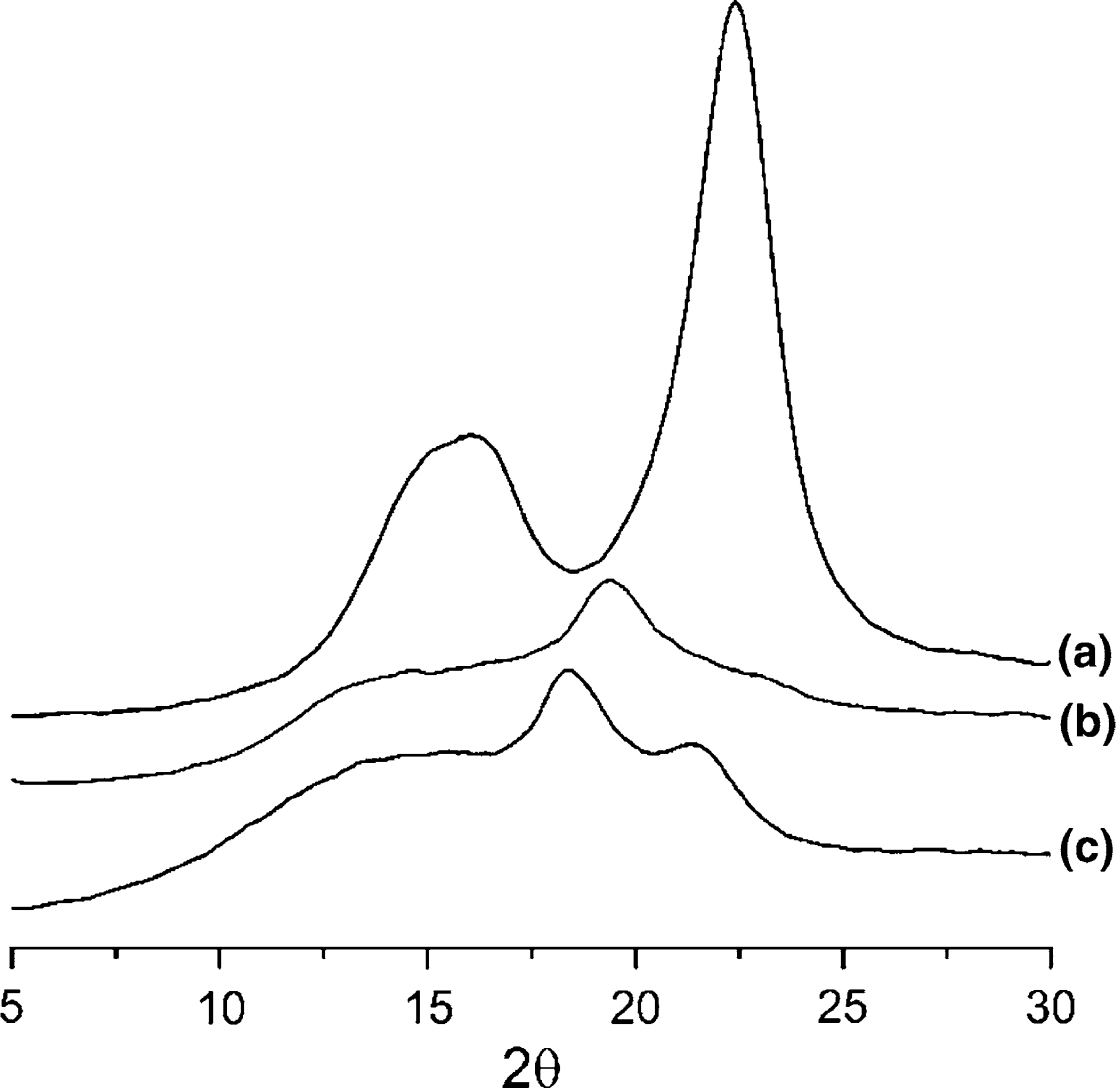
XRD patterns of **a** CC, **b** pure PVA film and **c** 9 % CC and PVA composite

The X-ray diffraction patterns of the CC, pure PVA film, and their composites are shown in Fig. 3. Mwaikambo and Ansell (2002) observed that the crystallinity degree of jute fiber was 71 %. The crystallinity index of the CC was found as 74.9 %. The increased crystallinity degree that is imparted to the CC is probably due to acid hydrolysis. The higher crystallinity is associated with the higher tensile strength of the micro-fibrils. On the other hand, PVA exhibits a broad peak at 2*θ* = 19.0° and can be considered as less crystalline in nature than that of the CC. The peak at 2*θ* = 22° was sharper in the composite than that of the pure PVA film and this indicates the higher degree of crystallinity of the composites and the persistence of the I_β_ structure of cellulose even after the compounding process.

### Tensile properties

The tensile properties such as tensile strength (TS), elongation at break (Eb%), and tensile modulus (TM) of the composite and the pure PVA films are shown in Table 2. The tensile strength of neat PVA film was 17.2 MPa. The tensile strength of CC reinforced PVA films showed the highest value (43.9 MPa) at the loading of 9 wt.%. This value was 155 % higher than neat PVA film. However, the CC loading of more than wt.% to PVA matrix gradually decreased the tensile strength. The tensile strength of PVA films with 15 wt.% CC was 110 % compared to PVA film. The intermolecular forces between CC and the base PVA matrix may enhance the tensile strength of the PVA composite films. The TM has increased from 1,472 to 2,190 MPa for pure PVA film to 9 % CC containing composite. It was obvious that if TS was increased, then Eb% should be decreased and in this sample A yielded Eb 145 % and sample D gave 3.7 %. The enhanced TS and TM resulting from the composites demonstrated (a) the reinforcing effect of finely dispersed high-performance CC throughout the polymer matrix and (b) strong interaction between CC and PVA that ultimately enhances interfacial adhesion.

**Table 2:** Tensile properties and moisture content of the CC/PVA composites

Sample name	Tensile strength (MPa)	Standard deviation	Tensile modulus (MPa)	Standard deviation	Elongation at break (%)	Standard deviation	Moisture content (%)	Standard deviation
A	17.1	±0.77	1,470	±1.19	140	±1.96	8.9	±1.17
B	31.2	±1.10	1,786	±2.71	4.9	±0.97	12.2	±2.33
C	41.6	±0.44	1,867	±2.33	3.7	±0.14	12.3	±1.5
D	43.9	±0.89	2,190	±1.66	3.7	±0.33	12.7	±1.22
E	37.2	±1.9	2,134	±2.92	4.2	±1.11	12.2	±0.98
F	36.4	±1.2	1,735	±1.15	2.2	±0.66	17.8	±1.14

All data were analyzed by SPSS software, version 15 using one-way ANOVA analysis. The level of statistical significance was set at 5 % (*p* < 0.05)

### Thermal properties

**Fig. 4:**
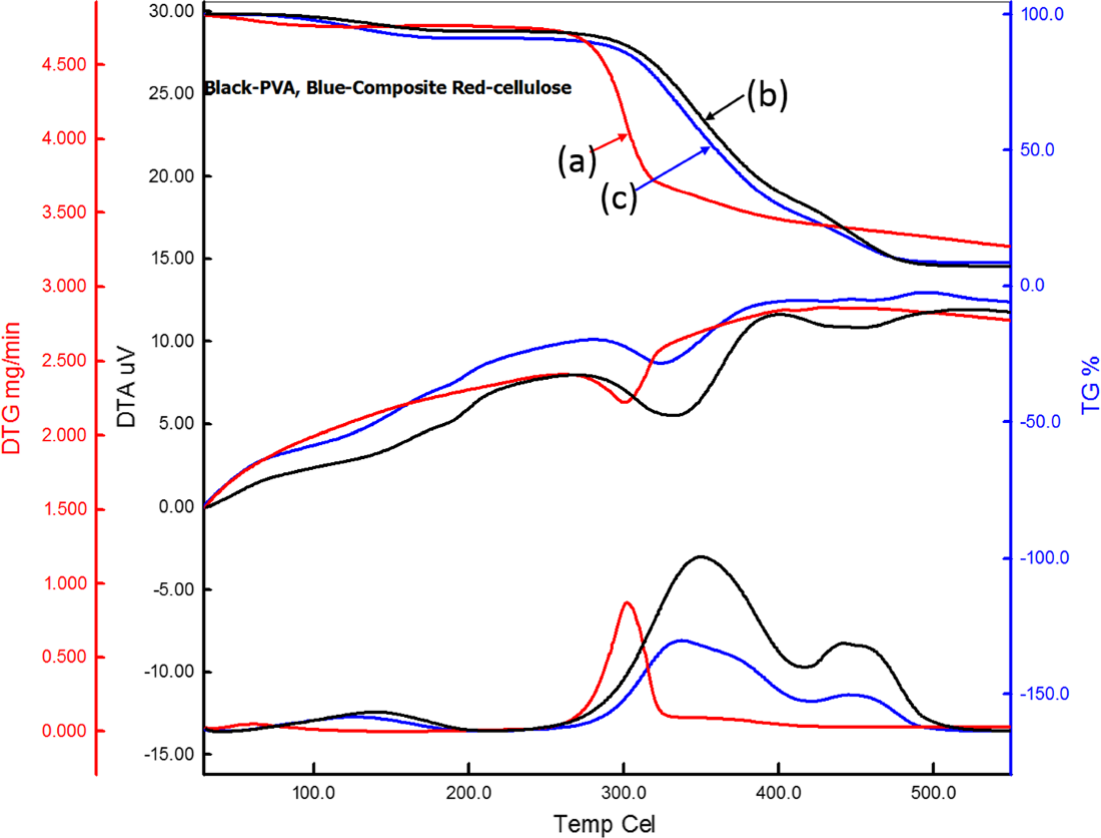
Comparison study of TG, DTA, DTG thermogram of **a** CC, **b** pure PVA film and **c** 9 % CC and PVA composite

 The results from the TG, DTG and DTA of CC, PVA and the composites are presented in Fig. 4. From TG analysis of CC, it was observed that these are thermally stable in the region below 280 °C. The initial weight loss of 3.9 % of CC at temperature (100–150 °C) was due to the evaporation of the adsorbed moisture. The temperature at onset and the maximum slope (50 % degradation) are 283 and 302 °C, respectively, for CC and the total degradation was 82 % up to the final temperature of 550 °C. The DTA curve shows two endothermic peaks at 301 and 413 °C due to thermal degradation. The DTG curve shows three types of degradation at 59, 302 and 374 °C. The maximum degradation occured at 302 °C with the rate of 0.872 mg min^−1.^ The main degradation at 302 °C for CC was due to depolymerization, dehydration, and decomposition of hydroxyl units followed by formation of char, while the degradation above this temperature can be described by the oxidation and breakdown of the char to low molecular weight gaseous products. Note that the crystallinity of the CC/PVA composite was lower than that of pure CC. The thermal degradation temperature of the CC/PVA composite should be shifted to a lower temperature. The reason might be due to the formation of a strong intermolecular reaction between CC and PVA, which can improve cohesive energy resulting in a higher thermal stability.

Thermogravimetry of the composite showed 8.7 % initial loss due to moisture and the onset temperature, and the maximum slope (50 % degradation) obtained are at 301 and 361 °C. The total degradation of 9 % CC and PVA composite was found to be around 90 %, which was slightly lower than that of the pure PVA film (92 %). The DTA curve shows five endothermic peaks at 126, 190, 325, 430, and 463 °C due to thermal degradation of composite materials; i.e., CC and PVA . The DTG curve shows three exothermic peaks of degradation at 124, 336 and 444 °C. The maximum degradation occurs at 336 °C with the rate of 0.610 mg min^−1^.

### Thermo-mechanical analysis

The TMA thermograms of pure PVA film and its composites with 9 % and 15 % CC are shown in Figure S1 (in the supporting information). It was observed that the softening of pure PVA film started at around 48 °C and it continues up to around 216 °C followed by the expansion which again continues up to 243 °C and finally melts down completely. However, the composite with 9 % CC shows the softening at around 35 °C and it continues up to 203 °C, then it started to expand and again continues up to 241 °C. Interestingly, it was noticed that the composite with 15 % CC softens at a higher temp (42 °C) and continues up to 203 °C, then it started to expand till 229 °C. The TMA data demonstrated that the softening and melting point of pure PVA does not differ significantly even after the fabrication of composites with CC.

### Water uptake and moisture content analysis

Both pure PVA film and the c composite are hydrophilic in nature and so get dissolved into water in a very short time as shown in Fig. S2 in the supporting information. It is observed that the composite film first swells in water and then starts to dissolve after 30 min of immersion in water. This has occurred because PVA has more –OH group than cellulose, that increases the polarity and hydrophilicity of the composite and, hence, makes it dissolve. The hydroxyl groups of PVA and cellulose can easily form hydrogen bonding with water, thus, this initiates the degradation. The moisture content analysis results of different films are shown in Table 2. It is found that the highest moisture content (18 %) was shown by 9 % CC containing composite and a least moisture content (9 %) by pure PVA film.

### Antimicrobial sensitivity and in vitro cytotoxicity study

The antimicrobial sensitivity test for the composites had a problem in that the PVA matrix melted down at the autoclave temperature of 37 °C. Though PVA films suffered from the problem of melting, after careful observation, it was found that samples having 6 % and 9 % CC in the composites (as shown in Figure S3 in the supporting information) showed a antimicrobial effect as clear zones (13 mm and 16 mm for *Escherichia coli* and 11 mm and 15 mm for *Bacillus subtilis*, respectively) of inhibition.

One method to evaluate cell and tissue response is to measure in vitro cytotoxicity, or its quality of being toxic to cells. Cell toxicity was determined by cell lysis (death) or the inhibition of cell proliferation. Prior to investigating a material in vivo, cytotoxicity can provide insight for any potential issues with the local tissue response. PVA film and the composite films dissolved in artificial sea water in which nauplius were inoculated. The number of deaths was the highest for the composite with 9 % CC and then decreases slightly with increased CC concentration as shown in Table 3. It may be due to three reasons: (a) the CC may have a cytotoxic effect, (b) dissolved oxygen concentration of the saline water may be decreased with time and (c) a layer of CC and PVA may be formed on the gills’ of nauplii. The results suggested that the possible reason of nauplii death was not toxicity as the number of death was nil for lower CC concentrations (3 % in the composite). Moreover, both CC and PVA are both biocompatible, thus, the best possible reason for the death of nauplii occurred due to the formation of CC and PVA layer on their gills. Lack of oxygen availability was also a fatal factor here because this viscous layer limits oxygen permeability through the gills.

**Table 3:** Mortality of Brine shrimp (*Artemia salina*) nauplii at different concentrations of CC/PVA composites

Sample name	Dose (mg/L)	No. of nauplii present after incubation	Mortality (%)
Positive control (*Vincristine sulphate*)	0.5	0	100
Negative control (artificial sea water)	–	10	0
A	0.2	10	0
B	0.2	10	0
C	0.2	9	10
D	0.2	5	50
E	0.2	7	30
F	0.2	6	40

## Conclusion

The detailed characteristics of the data of the CC/PVA composites can be concluded as follows:

- Cellulose crystals (micro and nano) were extracted from jute by hydrolysis with 40 % sulfuric acid using mechanical stirring for 10 h. After hydrolysis, the sample was centrifuged and freeze dried to obtain CC. FT-IR, XRD and SEM analysis confirmed the presence of microstructures of CC and some nanocrystals as well.
- CCs reinforced PVA composites were prepared by solvent casting and FTIR spectra confirmed that chemical binding occurred between PVA and CC molecules. This is evident in improvements of the compatibility, thermal properties and the mechanical properties of the composite.
- The composite sample D containing 9 % CC yielded the best mechanical, thermal, moisture resistance and antimicrobial properties.

Based on the above results, it can reasonably be concluded that CC/PVA composites have the potentiality to be used in biomedical purposes, and it may act to mimic the natural moist environment of a wound surface, which will eventually lead to accelerated wound healing.

## Electronic supplementary material

Below is the link to the electronic supplementary material.

Supplementary material 1 (DOCX 2139 kb)
