# Supplementary material for: Characterization of crystalline cellulose of jute reinforced poly (vinyl alcohol) (PVA) biocomposite film for potential biomedical applications
Source: Prog Biomater. 2014 Apr 3;3:23. doi: 10.1007/s40204-014-0023-x (PMC5151122; doi:10.1007/s40204-014-0023-x)
Supplement: Supplementary file 1 — Supplementary material 1 (DOCX 2139 kb) [file 40204_2014_23_MOESM1_ESM.docx]

**Supporting Materials**

**Characterization of crystalline cellulose of jute reinforced poly (vinyl alcohol) (PVA) biocomposite film for biomedical uses**

Mohammed Mizanur Rahman*, Sanjida Afrin and Papia Haque

*Department of Applied Chemistry and Chemical Engineering, University of Dhaka, Dhaka 1000, Bangladesh*

*Corresponding author

[Tel: +880-2-9661920-70/7392](Tel:+880-2-9661920-70/7392)

Fax: +880-2-861853

E-mail: [mizanur.rahman@du.ac.bd](mailto:mizanur.rahman@du.ac.bd)

Figure S1 a) TMA thermogram of pure PVA film

Figure S1 b) TMA thermogram of 6% cellulose reinforced PVA film

Figure S1 c) TMA Thermogram of 15% cellulose reinforced PVA film


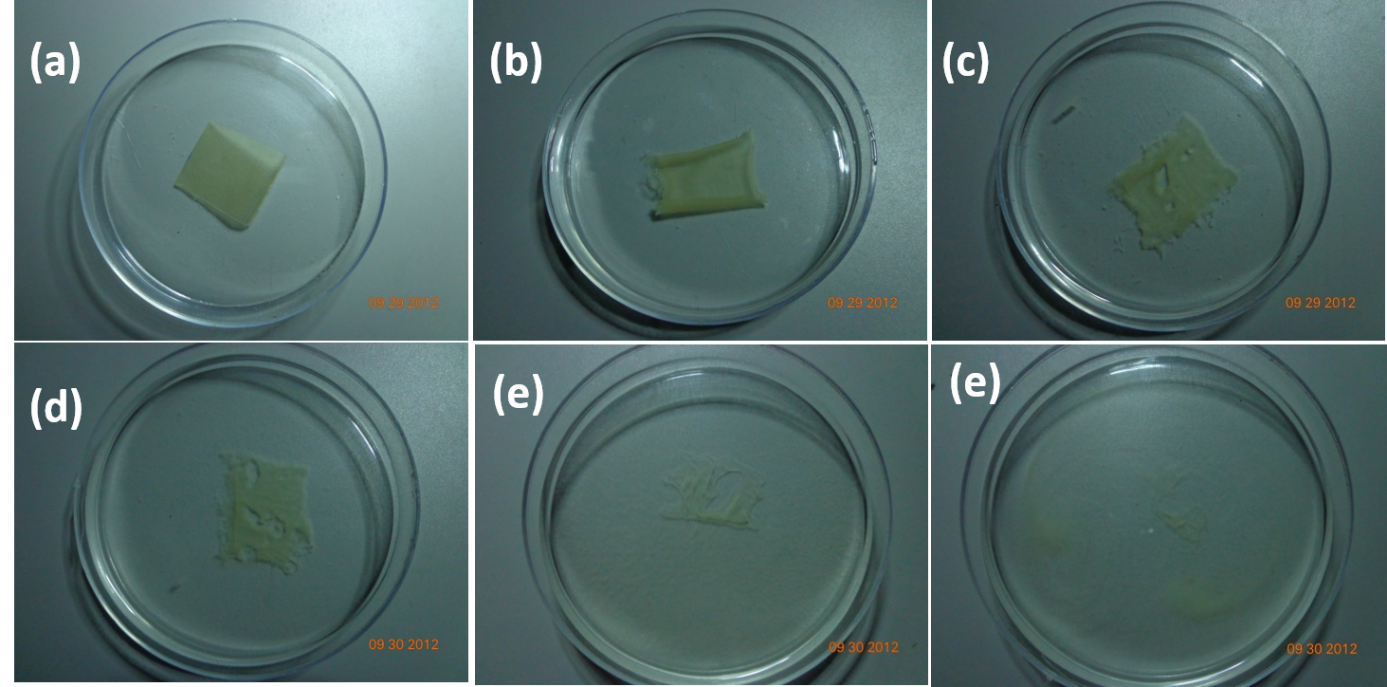


Figure S2. Water uptake of PVA-CC composite at different time of soaking in water; (a) no water, (b) after 15 min, (c) after 30 mins, (d) after 90 min, (e ) after 120 min and (f) after 180 min.


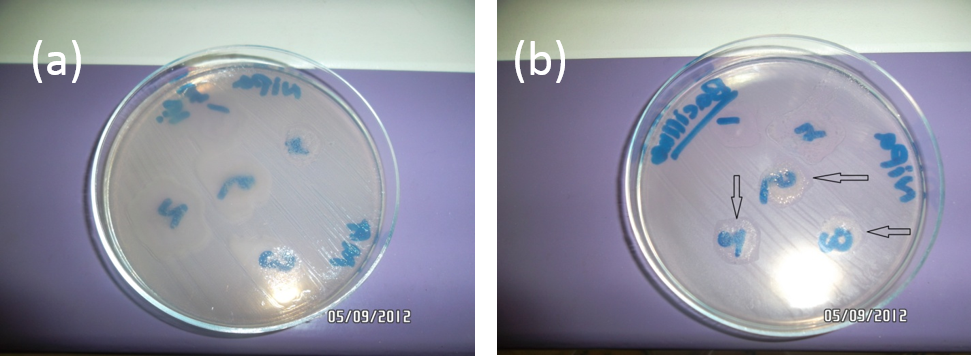


Figure S3. Anti-microbial activities of PVA-CC (9%) composite; (a)*E. coli*, (b) *Bacillus subtilis*

**
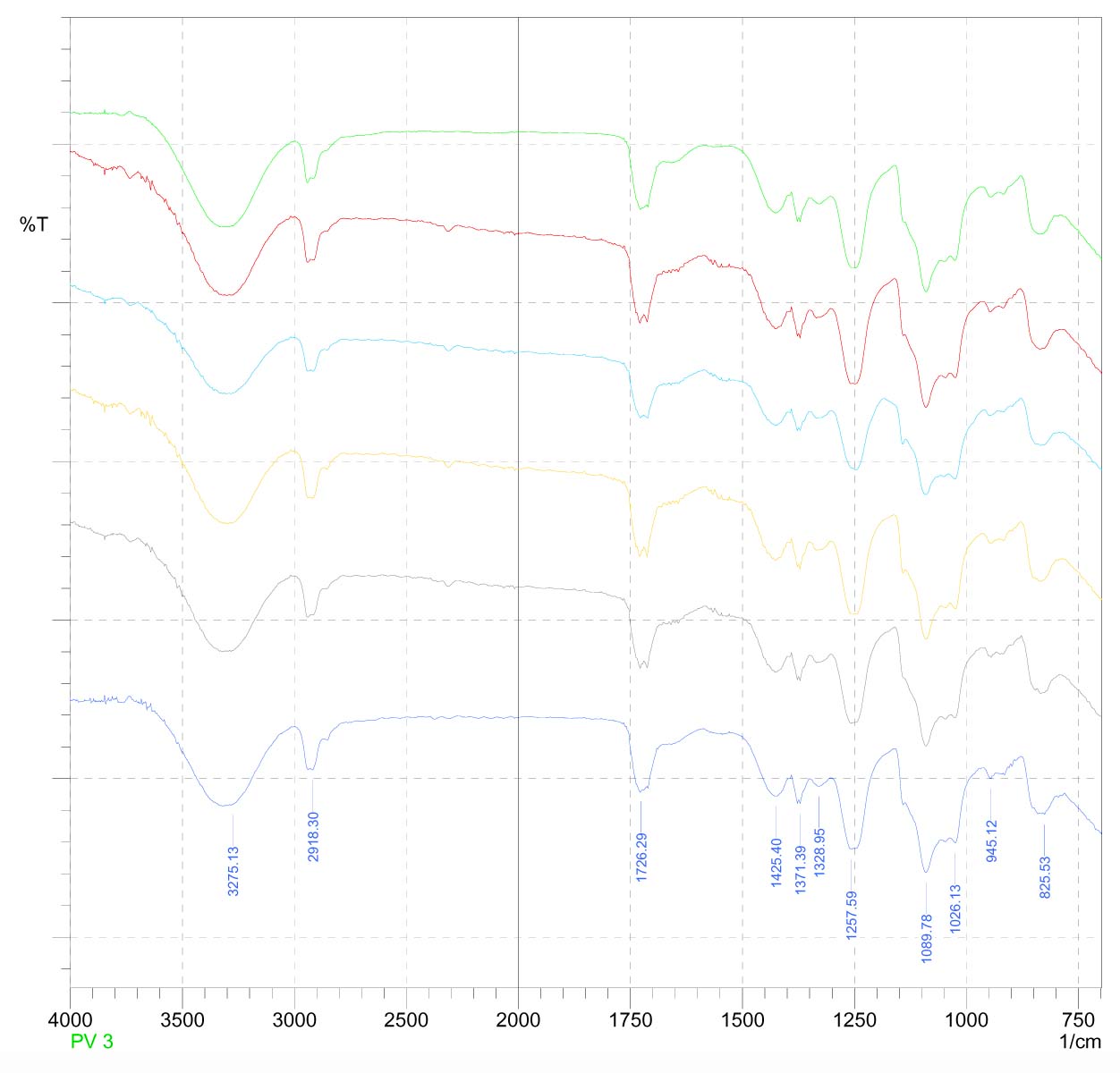
**

Fig S4. FTIR Spectrum of PVA/cellulose Composites (blue peak indicates PVA and the upper peaks indicate the composites)
